# Research progress in Pusher Syndrome after stroke

**DOI:** 10.3389/fneur.2025.1591872

**Published:** 2025-04-16

**Authors:** Huayong Wu, Haoyang Duan

**Affiliations:** ^1^College of Sports Science and Health, Harbin Sport University, Harbin, China; ^2^Department of Rehabilitation Medicine, First Hospital of Jilin University, Changchun, China

**Keywords:** Pusher Syndrome, stroke, rehabilitation, research progress, review

## Abstract

Post-stroke Pusher Syndrome is a postural control disorder. It is characterized by active tilting toward the hemiplegic side and resistance to correction. This significantly impacts patients’ motor function and quality of life. Its incidence varies greatly due to different research designs and assessment criteria. Literature reports an incidence ranging from 5% to 63%, and the incidence in patients with right brain damage (17.4%) is much higher than that in patients with left brain damage (9.5%). Etiological studies indicate that damage to the parietal lobe, thalamus, insula, and postcentral gyrus is the main pathological basis. The key mechanism is the interruption of thalamocortical connections. Typical clinical manifestations include trunk tilting in supine position, asymmetric weight-bearing in sitting, weight shift in standing, and impaired weight transfer during gait. Patients often have unilateral spatial neglect, which exacerbates balance disorders. Prognosis shows about 90% of patients recover within 6 months, but 10% to 15% may have long-term symptoms. Early rehabilitation intervention can significantly improve functional outcomes. This article comprehensively reviews the nomenclature, incidence, etiology, lesion sites, clinical manifestations, and prognosis of Pusher Syndrome, providing a research foundation for future studies on post-stroke Pusher Syndrome.

## 1 Introduction

Stroke is a major disease threatening human health (1–3). Its high incidence and mortality cause great pain to patients and impose a heavy economic and psychological burden on their families (53). Among post-stroke complications, balance and posture control disorders are common, significantly reducing patients’ daily living abilities and quality of life. Pusher Syndrome, a typical post-stroke posture control disorder, is characterized by patients actively tilting their bodies toward the hemiplegic side and resisting correction (4). Epidemiological studies show it has a high incidence among stroke patients and is a key factor affecting functional rehabilitation and quality of life.

In recent years, with the deepening of neuroscience and rehabilitation medicine research, Pusher Syndrome has become a hot topic in rehabilitation medicine (5–7). This imposes higher diagnostic and therapeutic demands on clinical professionals. Therefore, exploring its development is theoretically and practically significant. This article aims to comprehensively review and comment on the research progress of post-stroke Pusher Syndrome.

## 2 The origin and naming of Pusher Syndrome

The concept and naming of Pusher Syndrome originated in 1985 when Davies first systematically proposed and described it. Davies emphasized in his pioneering research that Pusher Syndrome is a unique postural control disorder in stroke patients. Through clinical observation, he found that patients in sitting, standing, or walking positions actively use their unaffected limbs to push their bodies toward the unaffected side, causing the center of gravity to abnormally shift to the hemiplegic side. They also strongly resist any external attempts to correct their posture. This abnormal movement pattern significantly impedes patients’ ability to perform daily activities, especially in transferring from bed to chair, maintaining standing balance, walking, and self-care activities like dressing and washing (8). Davies’ description not only clarified the core clinical features of Pusher Syndrome but also laid the theoretical foundation for subsequent research on pathogenesis and rehabilitation interventions.

In early literature, terms describing the “Pusher Syndrome” phenomenon were inconsistent. Pedersen et al. (9) first introduced the term “ipsilateral pushing” to refer to patients’ active pushing toward the hemiplegic side (the side opposite to the brain injury). In 2000, the term “contraversive pushing” was proposed to describe patients’ behavior of pushing their bodies toward the side opposite to the brain injury (10). D’Aquila et al. (11) defined “lateropulsion” as a postural control disorder where patients have the illusion of remaining upright when their bodies tilt to the hemiplegic side and resist postural correction. Karnath et al. interpreted “lateropulsion” differently, specifying it as a tendency to fall toward the same side as the brain injury, common in patients with lateral medullary syndrome. They also defined “pushing behavior” as patients pushing their bodies toward the hemiplegic side, accompanied by extension and abduction of the non-paralyzed limbs and resistance to passive postural correction. This description essentially refers to the same clinical phenomenon as “lateropulsion” proposed by D’Aquila et al. (12).

The term “Pusher Syndrome” comes from patients’ unique Clinical manifestations: when walking or standing, they exhibit a noticeable “pushing” motion toward the hemiplegic side, vividly called “Pusher.” In this review, we will uniformly use the term “Pusher Syndrome,” whose definition includes all behaviors of actively pushing the body toward the hemiplegic side, such as “ipsilateral pushing,” “contraversive pushing,” “lateropulsion,” “pushing behavior,” and “tilting syndrome.” Choosing this standardized term not only intuitively reflects patients’ clinical characteristics but also helps unify academic discussions and promote in-depth research and academic exchange in this field.

## 3 Prevalence of Pusher Syndrome

The reported incidence of Pusher Syndrome varies significantly due to differences in research design, sample characteristics, and assessment criteria. Davies first described it in 1985 with an incidence of about 25% in stroke patients (8). In 1996, the Copenhagen Stroke Study found an incidence of 5%–10% (9). Other studies indicate an overall incidence of 5–20% in stroke patients (13, 14), particularly in those with recurrent or severe brain injury. Notably, some studies have reported an incidence as high as 63%, often in patients with severe neurological impairment (15).

Yang et al. (16) assessed 48 stroke patients, finding 56.3% had mild Pusher Syndrome and 16.7% severe at admission. By discharge, 47.9% remained mild, 43.8% had no symptoms, and no severe cases remained.

Abe et al. (13) assessed 1,660 acute stroke patients with a standardized contralateral tilt scale, finding 154 (9.4%) exhibited tilting behavior. Of these, 97 (17.4%) had right-brain damage, and 57 (9.5%) had left-brain damage. This shows right-brain damage patients are at higher risk of tilting behavior.

It should be noted that the incidence of Pusher Syndrome can be influenced by patient age, gender, and the timeliness and effectiveness of rehabilitation. Therefore, future research should focus on well-designed, adequately powered multicenter studies to better define the epidemiology of Pusher Syndrome and confirm these findings.

## 4 The etiology of Pusher Syndrome

Pusher Syndrome is a complex neurological disorder with no fully understood cause. Current research indicates stroke is the primary cause, especially when it affects key brain areas for spatial orientation and body awareness, such as the parietal lobe, thalamus, or posterior thalamus. Damage to these regions can lead to typical Pusher Syndrome manifestations (17, 18).

Brain injury from traumatic brain injury, which results from external force to the head, can disrupt brain function and increase the risk of Pusher Syndrome (19). Post-surgical brain complications, like local tissue damage or inflammation, may also trigger it.

In central nervous system diseases, conditions like multiple sclerosis, brain tumors, or CNS infections can be associated with Pusher Syndrome if they affect brain regions related to posture and balance. These diseases can impact brain function through various mechanisms, including direct neuronal damage, inflammation, or tumor-related brain compression (19). It is important to note that Pusher Syndrome likely results from multiple factors interacting, with significant individual variation. Therefore, the specific cause can vary from patient to patient.

## 5 Lesion sites of Pusher Syndrome

Since Davies first described Pusher Syndrome, many studies have explored the link between brain damage sites and the syndrome (20–22). However, no specific brain area has been clearly identified as its direct cause. This might be because Pusher Syndrome involves complex interactions among multiple neural networks, not just isolated damage to a single area.

Research shows that patients with right hemisphere lesions have a higher incidence of Pusher Syndrome (17.4%) compared to those with left hemisphere lesions (9.5%) (13). Dieterich et al. (23) supported this, noting the right hemisphere’s special role in spatial orientation, memory, and navigation. Damage to these areas increases the risk of posture and spatial perception problems, thus raising the likelihood of Pusher Syndrome.

Baier et al. (18) found that in right hemisphere damage, the posterior insula, tectal area, and superior temporal gyrus are significantly associated with Pusher Syndrome, possibly being key parts of the sensory vestibular cortical network for balance and spatial orientation. In left hemisphere damage, the anterior insula, tectal area, and fibers in the internal capsule projecting to the lateral thalamus are linked to the syndrome, possibly directly related to posture control or processing vestibular balance information.

Karnath et al. (10) conducted a prospective study on 40 thalamic stroke patients, finding that right thalamic damage led to spatial neglect, while left thalamic damage resulted in aphasia. This highlights the thalamus’s importance in posture control and spatial perception. Another study revealed significant damage in the ventral posterolateral areas of the thalamus in Pusher Syndrome patients, especially those with thalamic hemorrhage, and this was confirmed in a three-year follow-up, strengthening the link between Pusher Syndrome and thalamic damage (24).

Karnath later noted that Pusher Syndrome is mainly related to posterior thalamic damage, with some cases also involving the insula and postcentral gyrus (12). He also found that damage to other cortical and subcortical regions, such as the insular cortex or postcentral gyrus, could cause Pusher Syndrome (25).

Johannsen et al. (26) studied 45 patients with acute unilateral cerebral cortical damage (without thalamic damage) exhibiting Pusher Syndrome behaviors. Results showed that brain area damage associated with these behaviors mainly focused on the insular cortex and postcentral gyrus, with significant overlap. Lee et al. (17) expanded on this, identifying significant associations between Pusher Syndrome and multiple specific regions in the right hemisphere, including the precentral gyrus, postcentral gyrus, inferior frontal gyrus, insula, and inferior parietal lobule.

Santos-Pontelli et al. (27) used advanced neuroimaging to analyze 31 Pusher Syndrome patients’ brains, finding the parietal lobe and thalamus as the most frequently affected regions. Later, using MRI perfusion imaging, they revealed significant hypoperfusion in the inferior frontal gyrus, middle temporal gyrus, and inferior parietal lobule. This suggests that hypoperfusion in these areas may directly impair posture control, indicating they could be key neuroanatomical bases for Pusher Syndrome (28).

Some studies link Pusher Syndrome to severe unilateral spatial neglect after parietal lobe damage, considering it a crucial pathological basis for Pusher behavior. However, other studies propose that posterior limb of the internal capsule damage might contribute to Pusher Syndrome by affecting sensory signal pathways (10). Jang et al. (20), in a diffusion tensor tractography study on intracerebral hemorrhage patients, found that as medial lemniscus nerve fibers repaired, Pusher Syndrome symptoms improved, indicating the medial lemniscus’s potential role in symptom alleviation.

Babyar et al. (29), using perfusion-weighted imaging, found hypoperfusion mainly in the inferior parietal lobe, from Brodmann area 2 in the postcentral gyrus to Brodmann area 40 in the inferior parietal lobule. This further links hypoperfusion in this region to Pusher Syndrome. Research indicates that Pusher Syndrome is typically associated with unilateral lesions in areas like the thalamic posterior nucleus, posterior insular cortex, superior temporal gyrus, postcentral gyrus, and inferior parietal lobule, which are key parts of the multisensory cortical network (e.g., the central vestibular system).

Recent studies using diffusion tensor imaging have explored vestibular connectivity, revealing close functional and structural links between vestibular nuclei and the central vestibular system. These interactions are crucial for maintaining balance and spatial orientation (23). Yeo et al. (30) found that when vestibular projection pathways are damaged, patients exhibit severe Pusher Syndrome symptoms, which improve as these pathways recover.

Rosenzopf et al. (31) analyzed functional and structural disconnection in 124 stroke patients to investigate Pusher Syndrome’s neural mechanisms. Using lesion network symptom mapping, they found functional deactivation in distant cortical areas (cerebellum, frontal, parietal, and temporal lobes) in thalamus-damaged Pusher Syndrome patients. Notably, this deactivation wasn’t seen in cortex-only stroke patients, and there was no evidence of convergence between thalamic and cortical damage in shared functional networks. Structural disconnection analysis confirmed disrupted connections between the posterior thalamus and temporal, precentral, postcentral, and paracentral regions. Tractography showed that in cortex-damaged Pusher Syndrome patients, critical white matter damage disrupted connections between the posterior thalamus and these regions, rather than the cortex damage itself causing the syndrome. This provides the first direct evidence of thalamocortical connectivity’s role in Pusher Syndrome, with the research team positing that the syndrome likely results from direct damage to the posterior thalamus or its connecting pathways.

## 6 The characteristic manifestations of Pusher Syndrome

Pusher Syndrome, a common post-stroke postural control disorder, has distinct and consistent clinical manifestations (32).

In supine position, patients exhibit a typical tilt toward the hemiplegic side due to trunk asymmetry, shortening the affected trunk. This compromises comfort and limits self-turning and movement. To compensate, patients often turn their head to the healthy side, opposite to the trunk tilt (33–35). Notably, even at rest, patients may experience vertical spatial perception errors, gripping the healthy bedrail to prevent falls (16, 36, 37).

In sitting position, patients display postural abnormalities from hemiplegic side hypertonia and trunk asymmetry: the affected trunk tilts, the buttocks bear weight, and the healthy trunk shortens, creating a pronounced asymmetry. This increases the difficulty of maintaining a seated position and fall risk. For compensation, patients turn their head to the healthy side, counteracting the trunk tilt but worsening sitting balance. During dynamic activities like transfers, patients struggle due to hemiplegic side resistance and balance issues, especially when moving to the healthy side. Some patients also have unilateral spatial neglect, exacerbating balance problems and risks (21, 38).

In standing position, patients show significant weight shift to the affected side, tilting the trunk there and struggling to maintain upright balance. Some lean backward, needing therapist or device support. Kinesiologically, the affected lower limb has flexor hypertonia and extensor weakness, limiting weight-bearing. Patients have difficulty initiating gait due to impaired weight shift to the healthy side. Notably, those with spatial neglect may have additional balance and environmental judgment issues (39, 40). This instability causes significant fear of falling, leading to over-reliance on others or supports (32).

During gait cycle, patients have significant weight transfer deficits, struggling to shift weight from the affected to healthy side, reducing gait stability and causing a lateral lean. This mainly stems from affected lower limb flexor-extensor imbalance: hypertonia and weakness respectively, maintaining a flexed posture and impairing weight-bearing. At gait initiation, the healthy lower limb has stepping difficulty, shorter support phase, and smaller step length due to limited weight shift. The affected lower limb lacks sufficient extensor activation for antigravity support during stance phase, increasing fall risk. To compensate, patients show typical postural adjustments: turning the head to the healthy side for visual balance and tilting the trunk to the affected side to reduce weight on it (41).

These clinical manifestations highlight the multidimensional postural control deficits in Pusher Syndrome patients post-stroke. These deficits severely impact motor functions like position changes, sitting balance, standing stability, and gait control, negatively affecting daily activities, social participation, and quality of life. Moreover, this unique postural disorder can prolong rehabilitation, increase its difficulty, and influence overall prognosis (7, 42). Thus, understanding these clinical features is crucial for developing targeted rehabilitation strategies and improving patient outcomes.

## 7 Prognosis of Pusher Syndrome

Research on Pusher Syndrome prognosis presents diverse findings. Evidence suggests it significantly delays rehabilitation compared to other post-stroke disorders, particularly in the early stage, with the delay lasting weeks to months, yet generally not affecting final functional recovery (15, 43). Prognostic differences are mainly attributed to etiological factors [e.g., traumatic brain injury or brain tumors, which often require longer recovery (19)], lesion site impact [right-brain-damaged patients usually recover slower than left-brain-damaged ones (13)], and comorbidities [patients with proprioceptive or visual-spatial disorders typically need longer rehabilitation (44)]. Danells et al. (18) found a significant link between Pusher Syndrome duration and unilateral spatial neglect in their longitudinal study of 39 patients.

Long-term prognosis research results are inconsistent. Karnath’s prospective study shows about 90% of patients recover nearly fully within 6 months, with no significant long-term functional impact (45). However, clinical observations indicate 10%–15% of patients still have symptoms 2 years post-stroke, which may continuously affect daily activities (43).

Early identification and intervention are crucial for improving prognosis. Systematic rehabilitation significantly enhances postural control and balance, shown in: improved daily activity ability, enhanced self-care ability, better quality of life, reduced complication rate (46, 47). Thus, establishing an early diagnosis system and creating individualized rehabilitation plans are crucial for optimizing Pusher Syndrome patient prognosis.

## 8 Advances in the treatment of Pusher Syndrome

Transcranial direct current stimulation (tDCS) is a non-invasive brain stimulation technique that modulates cortical neuronal activity by applying weak direct current to the scalp. Its mechanism is based on changing neuronal resting membrane potential: anodal stimulation increases neuronal excitability, while cathodal stimulation decreases it. In treating Pusher Syndrome, tDCS mainly modulates cortical areas related to balance control. Studies show that tDCS applied to the scalp or mastoid can effectively improve post-stroke limb motor and cognitive dysfunction, positively affecting posture control and significantly promoting patient rehabilitation (4, 48, 49).

Transcranial magnetic stimulation (TMS) is another non-invasive brain stimulation technique that induces cortical current changes via a magnetic field generated through the scalp, modulating neuronal excitability. Its effects depend on stimulation frequency and intensity: high frequency enhances neuronal excitability, while low frequency reduces it. In Pusher Syndrome treatment, TMS mainly improves motor function and balance control. By stimulating specific brain regions like the motor and vestibular cortices, TMS promotes neuroplasticity, significantly enhancing motor ability and posture control. Notably, repetitive TMS is recognized by the American Heart Association/American Stroke Association as an effective method for improving Pusher Syndrome-related neglect symptoms, with evidence level IIb and grade B (50).

Brain stimulation techniques show broad prospects in treating Pusher Syndrome. They precisely modulate cortical excitability, effectively improving motor function and balance control, offering new rehabilitation ideas. However, further research is needed on optimal stimulation parameters, target area selection, and long-term efficacy. Future studies should focus on developing individualized treatment strategies and combined protocols of brain stimulation with traditional rehabilitation training for optimal Pusher Syndrome rehabilitation.

Studies indicate that virtual reality technology offers continuous visual feedback to patients, guiding correct posture and promoting central nervous system restructure (54). The research of Kim et al. (41) confirms that virtual reality, through real-time visual feedback, corrects abnormal postures and accelerates neuroplasticity, enhancing rehabilitation outcomes. Combining visual guidance with core stability training also effectively improves tilting posture and enhances body vertical perception.

Research further explores the combined use of virtual reality and robotics. This technology provides highly repetitive training and allows for early upright posture training via harness assistance. Although more effective than conventional physical therapy combined with visual feedback, it remains unclear whether real-time therapist guidance and correction are included during robotic-assisted treatment (51, 52).

Both traditional mirror visual feedback and computer-based virtual reality technologies have positively influenced posture control, improving quality of life. Despite the advantages of computer visual feedback systems in therapeutic effects and precision, their high equipment costs limit their widespread use in clinical and home settings.

## 9 Summary

This article comprehensively reviews research on Pusher Syndrome post-stroke, covering its origin, naming, incidence, etiology, lesion sites, clinical manifestations, and prognosis. Pusher Syndrome is a complex neurological disorder where patients actively tilt toward the hemiplegic side and resist postural correction, significantly impacting motor function and quality of life. Its causes are diverse, mainly related to damage of the multisensory cortical network from stroke, such as in the parietal lobe, thalamus, insula, and postcentral gyrus. Studies show a higher incidence in patients with right-brain damage than left-brain damage, with different brain injuries linked to varying clinical manifestations and prognoses. Typical manifestations include trunk tilting in supine position, asymmetric weight-bearing in sitting, weight shift in standing, and impaired weight transfer during gait. For prognosis, most patients recover within 6 months post-stroke, but some have long-term symptoms. Early rehabilitation significantly improves functional outcomes. Future research should clarify its pathological mechanisms to refine diagnostic and therapeutic strategies.
